# Cocreating the Principles of Compassionate Virtual Care With South Asian Immigrants: Protocol for a Qualitative Study

**DOI:** 10.2196/103397

**Published:** 2026-08-27

**Authors:** Saleema Allana, Salsabela Razaq, Thandiwe Denga, Aaron Folahan, Philip Olumilua, Victoria Smye, Richard Booth, Allison Crawford

**Affiliations:** 1 Western University London, ON Canada; 2 Black Youth Mentorship and Leadership Program University of Calgary Calgary, AB Canada; 3 Centre for Addiction and Mental Health Toronto, ON Canada

**Keywords:** South Asian, immigrants, cocreation, compassionate care, virtual care

## Abstract

**Background:**

Virtual health care has become increasingly popular worldwide since the COVID-19 pandemic. Immigrant populations in Canada face significant barriers to, and disparities in, access to and use of virtual care. South Asian immigrants in particular often face intersectional impediments to health care access. Compassionate and equitable health care is a fundamental patient right and should be provided at all levels of the health care system; however, patients frequently report a lack of compassionate, respectful, and caring health services.

**Objective:**

There is a large gap in studies conducted among South Asian immigrants, particularly regarding their access to and use of virtual care and their perceptions of compassionate virtual care. This study aims to address this gap by exploring how South Asian immigrants in Canada understand and define compassion, trust, and equity within the context of virtual care.

**Methods:**

This research will be underpinned by the framework on digital intersections with compassionate care. This patient- and community-partnered cocreation research is informed by a qualitative, descriptive, participatory approach. This research will be conducted in three phases: (1) a scoping review to explore this emerging area of research; (2) one-on-one qualitative interviews with South Asian patients to examine compassionate, equitable, trusting, and productive patient–health care professional relationships in the context of virtual care; and (3) collective virtual meetings to cocreate principles of compassionate and equitable virtual care. This participatory research will be guided by the SPOR Patient Engagement Framework to ensure meaningful engagement throughout the study. Additionally, the three co’s framework will guide the patient partnership. South Asian patients will be recruited from the community via collaboration with community organizations and Facebook groups. Purposive sampling will be used for an estimated sample size of 15 to 20 patients. An inductive content analysis will be applied. The data will be read thoroughly, and the text will be coded based on the content within the data. The codes will be sorted into categories to formulate themes.

**Results:**

The findings are expected to have implications for practice, research, and education. The study was funded in June 2023; however, an extension was granted to accommodate the principal investigator’s maternity leave and facilitate completion of the study. Data collection is projected to take place between June and August 2026. As of June 17, 2026, two participants have been recruited. Data analysis will commence in July 2026, following the completion of initial interviews, with findings expected to be published in winter or spring 2027.

**Conclusions:**

This study fills a research gap regarding South Asian immigrants’ access to, use of, and perceptions of compassionate virtual care to promote its adoption.

**International Registered Report Identifier (IRRID):**

PRR1-10.2196/103397

## Introduction

### Background

Canada is characterized by considerable cultural heterogeneity, with diasporas from around the world calling the country home [1]. Acknowledging these pluralistic values, equity in the opportunities available to these varying ideations and beliefs becomes imperative. As of 2021, immigrants are documented to account for 23% of Canada’s total population [2]; of this 23%, South Asians make up 7.1% [3]. Furthermore, recent official population projections foresee a sustained increase in the number of South Asians immigrating to Canada, with predictions ranging from 5 million to 6 million additional South Asian migrants by 2041 [4]; however, despite a substantial portion of the national population being South Asian, they continue to experience inequities in their access to and use of the Canadian health care system.

### Access to Care

South Asian immigrants often face intersectional impediments to health care access. Language barriers, cultural differences, and socioeconomic factors contribute to disparities in health care access among South Asian immigrants. For example, language barriers between patients and health care professionals necessitate the use of third-party translation, subsequently reducing the accuracy of communication of health diagnoses to South Asian patients [5,6]. Furthermore, a 2020 study by Turin et al [6] on the barriers to health care access faced by Bangladeshi men in Canada found that many of the research participants reported difficulty communicating their health concerns to health care providers due to insufficient time given to accommodate the strain of articulation in a second language. Cultural differences also serve to hinder South Asian immigrants’ access to appropriate health care; South Asian immigrant populations value cultural competence in their health care providers [7]. Cultural competence highlights the need for health care systems and providers to be aware of the intersections of identity, including language, cultural traditions, family preferences, socioeconomic conditions, and other dimensions of identity [8]. However, recurring reports of dissatisfaction among South Asians with their physicians’ general lack of knowledge regarding their culture highlights a disconnect between health care availability and access [7]. Additionally, lower socioeconomic status can contribute to restricted access to appropriate health care among South Asian immigrants [5].

### Use of Care

Equally important to ensuring access to health care services is ensuring their appropriate use by South Asian immigrants. Digital literacy, perceived usability, and acceptability of health care services play an important role in inhibiting health care use among South Asian immigrants. “Digital literacy” refers to one’s affinity for online interactions and the use of digital technology [9]; however, digital literacy requires a certain level of English proficiency, a level unattained by a considerable portion of the South Asian population in Canada—particularly older South Asians [10,11]. Moreover, assessing the utility of health care services must take into consideration the target population’s perceptions on how easy or difficult the services are to use, and how this dictates their choices [12]. In the study by Aldosari et al [10], several participants expressed a preference for radio over computers when accessing digital health information or services because computers were more difficult to operate than radios [11].

### Emergence of Virtual Care

The use of virtual health care has become increasingly popular globally since the COVID-19 pandemic [13-15]. For example, in China, the national health insurance agency began to cover the cost of virtual consultations in 2020, allowing the country’s physicians to consult with more than 100 patients a day through virtual platforms [16]. Adopting a similar approach, an additional 80 services were added to its telehealth coverage by the US Centers for Medicare and Medicaid Services [16]. A similar surge in the uptake of virtual care was seen in Canada, as virtual care went from making up just 1.8% of total primary care visits in the fourth quarter of 2019 to an all-time high of 70.2% in the second quarter of 2020 [17]. According to the Canadian Institute for Health Information, virtual care accounted for between 24% and 42% of services received by patients in 2020, a significant increase from the 2% to 11% the previous year [18]. Companies that aid in providing virtual care, such as Telus Health and WELL Health Technologies, have also contributed greatly to its growing popularity in Canada by providing patients with simple, convenient access to digital health services [19].

### Inequities in Virtual Care for South Asian Immigrants

Immigrant populations in Canada face significant barriers to and disparities in access and the use of virtual care. Language barriers, digital literacy, isolation, and health beliefs were noted to be significant barriers for older immigrant adults in Ontario [20]. During the pandemic, the lowest rates of virtual health visits were found to be among immigrants who were not proficient in French or English, as well as immigrants who solely spoke French [20]. This suggests disparities in the accessibility of health care services for those who do not speak the dominant language, which, regardless of the mode of communication, have the potential to translate to virtual care [20]. More specifically, immigrants to British Columbia aged >60 years with limited language ability in English or French were found to experience greater inequity in access to primary care [21].

Very little literature exists on the barriers affecting access to and use of virtual care among South Asian immigrants in Canada. In a participatory study of 197 South Asian community members in Surrey, British Columbia, Hyman et al [11] identified numerous barriers to accessing digital health tools among South Asian immigrants. Within a group of Punjabi-speaking South Asian immigrants, a limited use of health-managing technology was correlated to old age, limited language ability in English, low socioeconomic status, and being women [11]. A common theme found throughout the study was the importance of a strong literacy level when navigating digital health tools. A significant number of participants expressed that they found it challenging to read in any language, further limiting their use of digital health tools and technology in Canada [11]. Other barriers identified include a lack of trust in health sources provided digitally, preferences of a particular ethnic group, as well as caregiving responsibilities that limit time and access to these tools for South Asian immigrants [11].

### Compassionate and Equitable Care

Compassionate and equitable health care is a fundamental patient right and should be provided at all levels of the health care system [22]. However, patients frequently report a lack of compassionate, respectful, and caring health services [22]. A literature review identified a lack of compassion in health care delivery as well as insufficient training among health care workers to provide compassionate care to patients [22]. It is also important to understand that compassionate, equitable, and culturally safe care might have different meanings for various immigrant and equity-deserving groups [23]. A study was undertaken to understand what compassionate care means to the South Asian population as well as ways to enhance compassion provided by health care providers. The study concluded that the interpretation of compassion may vary depending on factors such as cultural and ethnic background [23].

Moreover, the meaning of compassionate and equitable care in the virtual context may also vary for people from different ethnic backgrounds, and their perceptions and experiences may affect their access to and use of virtual care [24]. A mixed-methods study was conducted to examine primary care physicians’ perspectives on the advantages and disadvantages of video-based care and the adequacy of the technology used to deliver it. This study demonstrated the need to enhance portal access for people with limited English-speaking capability. Another study was conducted to understand the steps needed to provide compassionate care through technology by exploring the perspectives of emergency physicians in the Greater Toronto Area [25]. The study identified strategies to improve equity in virtual urgent care, such as the implementation of community hubs for equity-deserving groups [26] to facilitate accessible and culturally safe care [25].

### Gap Statement and Study Purpose

Despite existing knowledge, there is still a large gap in studies conducted among South Asian immigrants’ access to and use of virtual care and how they perceive compassionate virtual care, illustrating the importance of the proposed study, which aims to address this gap by exploring how South Asian immigrants in Canada understand and define compassion, trust, and equity within the context of virtual care.

## Methods

### Conceptual Framework

This research will be underpinned by the framework on digital intersections with compassionate care. The framework on digital intersections with compassionate care encompasses six categories: (1) raising awareness of suffering; (2) mediating emotional responses to suffering; (3) creating a new means of responding; (4) providing a platform for education, coaching, and training around compassionate care in the digital ecosystem; (5) enabling compassionate actions; and (6) simulating or automating compassionate responses [27]. These categories will be used as a basis to understand compassion within virtual care for South Asian immigrants [27].

### Design

This patient- and community-partnered cocreation study is informed by a qualitative, descriptive, participatory research design that engages stakeholders who directly represent the interests of the target study population [28]. Qualitative descriptive participatory research is invaluable to this research as this approach will aid in uncovering and challenging an oppressive system to actively support the empowerment of disadvantaged communities, such as South Asian immigrants [29]. This research will be conducted in 3 phases. In the first phase, a scoping review has been conducted to explore this emerging area of research. The purpose of the scoping review was to inform the direction of this work. In the second phase, compassion, equity, trust, and a productive patient–health care professional relationship in the context of virtual care will be explored via one-on-one qualitative interviews with South Asian patients. The semistructured interview guide will be formulated based on 2 process-oriented tools exploring digital health compassion and equity [30,31].

These tools include the Telenursing Self-Assessment Tool and the Health Equity Impact Assessment-Digital Health supplement (HEIA-DH) [30,31]. While the Telenursing Self-Assessment Tool was originally designed for tele-nurses, it offers a valuable framework that will be integrated into the semistructured interviews to ensure patient-centered encounters [29]. The HEIA-DH supports the development of equitable digital health technologies, including virtual care [31]. The HEIA-DH is valuable to cocreate principles of compassionate and equitable virtual care for South Asian immigrants and will be used to guide the interview questions in this proposed research. The third phase seeks to cocreate the principles of compassionate and equitable virtual care through collective virtual meetings with the same group of South Asian patients who will participate in the second phase of the study. A maximum of 2 meetings will occur (1 individually and 1 in the group setting), and the study participants will be interviewed by the principal investigator and research assistants. The principles will be cocreated with study participants. The final principles will then be validated through participant feedback on the key findings that emerge from the interviews. This phase will use a participatory research approach, with South Asian immigrants and community partners or advisers equally involved in all study-related decision-making [32].

Participatory research is grounded in direct collaboration with people affected by the issue to generate actionable change [28]. The extent of participation varies between studies. This participatory research will be guided by the SPOR Patient Engagement Framework to ensure meaningful engagement throughout the study [33]. Consistent with the SPOR Patient Engagement Framework, meaningful involvement will be embedded throughout all stages of the research, including priority setting, study design, data collection, and dissemination of findings [33]. The SPOR Patient Engagement Framework outlines three core areas for engagement: (1) patient engagement in governance and decision-making, (2) capacity building for patient engagement, and (3) tools and resources [33]. Patient partners will be involved across the 3 core areas within this study [33]. Additionally, the three co’s framework will guide the patient partnership [34]. This framework involves the following phases: co-define (identifying the problem and goals of the group), co-design (solution to the problem), and co-refine (assessment of the solution). This participatory research will involve patient partners in all 3 phases outlined in the three co’s framework during the cocreation of the principles of compassionate and equitable virtual care [34].

### The Telenursing Self-Assessment Tool

The Telenursing Self-Assessment Tool comprises 5 sections [30]. Section 1, “opening the call,” determines if a nurse’s initial interaction sets the stage for a friendly and trustworthy interaction [30]. The second section, “listening and assessing,” evaluates active listening and the exploration of health problems [30]. The third section, “deﬁning diagnosis and goals, planning, and intervention,” assesses the information provided and collaboration on solutions [30]. The fourth section, “evaluation and conclusion,” evaluates the understanding of the information provided and addresses any further questions [30]. The fifth section, “overall issues,” evaluates various items necessary during the entire experience [30].

### Health Equity Impact Assessment and Digital Health Supplement

The HEIA-DH outlines 5 steps to promote equity in digital health care [31]. The first step, “scoping,” identifies the populations that are impacted by the digital health technology [31]. The second step, “potential impacts,” determines digital health technology’s positive and negative impacts. The third step, “mitigation,” is the exploration of strategies to increase positive impacts and reduce negative impacts [31]. The fourth step, “monitoring,” tracks the impacts and the respective mitigation strategies [31]. The fifth step, “dissemination,” suggests how the findings should be shared [31]. The HEIA-DH can be used in virtual care with various groups, including South Asian immigrants.

Patient engagement is essential to understand compassion in virtual care for South Asian immigrants [32]. A qualitative patient- and community-partnered approach will generate a person-centered understanding of compassion and equity in virtual care for South Asian immigrants [32,35]. Findings from the principal investigator’s prior research indicated that most virtual care initiatives have no specific considerations for ethnic minority groups or immigrants. This supports the exploration of compassion in virtual care for South Asian immigrants. This study uses a small purposive sample, as discussed in the Sample Recruitment section of this protocol paper. Participant recruitment is feasible due to the principal investigator’s established connections with community partners and the use of social media for participant outreach. The COREQ (Consolidated Criteria for Reporting Qualitative Research) checklist will be used for reporting the qualitative study, and the Guidance for Reporting Involvement of Patients and the Public 2 will be used to report for patient and public involvement in this study.

### Sample Recruitment

For this study, South Asian patients will be recruited through Facebook groups. However, to reach individuals with low digital literacy or those who speak limited English, South Asian patients will also be recruited from the community through the research team’s collaboration with community organizations. Purposive sampling will be used so that participants can be purposefully selected to inform an understanding of the central phenomenon the study aims to explore [28]. Purposive sampling will be used to recruit South Asian patients who have firsthand experience of using virtual care [28,36]. The estimated sample size for South Asian patients is 15 to 20 patients. In qualitative tradition, this sample size estimation is based on a reasonable sample to draw tentative conclusions [37,38]. As this is a qualitative study aimed at exploring the unique understandings of South Asian immigrants rather than making statistical inferences, a formal sample size calculation was not required [37,38].

### Data Collection and Analysis

An inductive content analysis (ICA) will be applied [39]. This analytical approach is particularly well suited for this study due to the limited existing data on the phenomenon under investigation, enabling a deeper understanding of this underexplored area [40]. As ICA is commonly applied to textual data, all interviews will be audio recorded and transcribed prior to analysis. The data will be read thoroughly, after which the text will be divided into condensed meaning units that will be assigned codes through the process of “coding” [40]. Coding involves identifying segments of text within the transcripts that share similarities [40]. The codes will not be predetermined; rather, they will be developed inductively based on the content of the data [40]. The coding process will be completed several times and refined based on comparisons between documents to ensure that no codes are missed [40]. The codes will be sorted into categories to formulate themes [39]. These themes will surface organically from the data instead of from preexisting theories or frameworks. Patient and community partners will be involved in discussions regarding the evolving themes.

### Ethical Considerations

Ethics approval for this study was granted by the Western Research Ethics Approval Board (project ID 126936). To ensure that consent is informed, all selected participants will receive an information letter via email prior to participation. Participants will be asked to review the document and confirm that they understand the study requirements and what will be expected of them. Participants will also be given a chance to ask questions and seek clarifications after reviewing the consent form, either via phone (the phone number of the principal investigator is provided in the consent form) or email. If satisfied, participants will provide electronic consent after reviewing the letter and confirm their willingness to participate. There are no foreseen risks, harms, or inconveniences arising from this study. All measures will be taken to maintain confidentiality, anonymity, and privacy of the participants and their data. To safeguard participant information, access to original recordings will be restricted to the study team, transcription of recordings will not include information that can identify the study participants (eg, participant names and/or other identifying information will be redacted), and recordings will be transcribed and assigned codes for identification; the recordings will not capture date and time. The recordings will be destroyed after the retention period specified in the ethics approval. Each participant who takes part in the study will receive an Amazon gift card as compensation for their time and effort.

## Results

The study was funded in June 2023; however, an extension was granted to accommodate the principal investigator’s maternity leave and facilitate completion of the study. Data collection is projected to take place between June and August 2026. As of June 17, 2026, two participants have been recruited. Data analysis will commence in July 2026, following the completion of initial interviews, with findings expected to be published in winter or spring 2027.

## Discussion

### Anticipated Findings

This study is expected to yield 2 primary outcomes. First, the findings will offer a nuanced understanding of how South Asian immigrants perceive equitable, trustworthy, and compassionate virtual care. As these perspectives are inherently subjective and shaped by cultural and ethnic backgrounds, defining these elements is essential for guiding future virtual care practice and research. Second, guiding principles for compassionate virtual care will be cocreated alongside South Asian immigrants to ensure future virtual health delivery is culturally relevant and tailored to their specific needs.

This qualitative research protocol paper highlights the disparities in health care that South Asian immigrants face routinely. This community- and patient-partnered research is guided by critical ethnographic approaches. Research backed by critical ethnography targets inequities as well as challenges the status quo [28]. The proposed study will be underpinned by the framework on digital intersections with compassionate care. This research will be administered in 3 phases. A scoping review was completed in the first phase to explore this emerging area of research and inform the direction of this work. The second phase will involve qualitative interviews with South Asian patients to examine compassionate, equitable, trusting, and productive patient-provider relationships in the context of virtual care. The interview guide will be formulated based on 2 process-oriented tools that target digital health compassion and equity: the Telenursing Self-Assessment Tool and HEIA-DH. The third phase of this research will involve cocreating the principles of compassionate and equitable virtual care with the South Asian patients who participated in the second phase. An ICA will be applied [39]. This analytical approach is specifically well suited for this study due to the limited existing data on the phenomenon under investigation. This provides a deeper understanding of this underexplored area [40]. Patient engagement is essential to understand compassion in virtual care for South Asian immigrants [32]. A qualitative patient- and community-partnered approach will bring about patient-centered understanding of compassion and equity in virtual care for South Asian immigrants [32,35].

### Strengths

The proposed study aims to fill a large gap in the existing literature through examining compassionate and equitable virtual care for South Asian immigrants. Compassionate care is heavily underresearched, even more so in virtual care and regarding South Asian immigrants, making this a novel topic. To our knowledge, this is the only study to date that has been planned to cocreate the principles of compassionate and equitable virtual care, specifically for the South Asian community, further contributing to the novelty and timeliness of this study [41]. The fact that this study is using cocreation with the South Asian community is a strength in itself. By working directly with members of the South Asian community during the research process, this study accurately exemplifies community-based participatory action research along with patient- and community-partnered research approach. Cocreation in research holds many benefits, including the ability to greatly impact society and provide a stage for voices in the community to be heard [42]. Not only does this method of research center on the lived experiences of the members of the communities it examines but it also recognizes the imbalances in power between researchers and community members and acknowledges that mutual trust must be built over time to bring sustainable change [42]. Additionally, the use of both the Telenursing Self-Assessment Tool and the HEIA-DH in the proposed study work together to strengthen the methodology of our research. The use of these tools offers structured frameworks to guide our research within this community, providing us with a consistent method of examining the quality and equity of the care provided.

### Limitations

The specific scope of this study involving South Asian immigrants means that it exclusively reflects the experiences regarding compassionate and equitable virtual care for this community alone. As such, the findings of this study may not be generalizable to other immigrant populations in Canada beyond the South Asian community, including those who may face similar barriers. Additionally, the nature of the subject that we are exploring—compassionate care—is inherently subjective. This suggests that perceptions of compassion can vary across communities when social and cultural contexts are taken into consideration. This further implies that the principles of compassionate and equitable virtual care that are cocreated in this study will only be reflective of the South Asian community. Qualitative research emphasizes the use of detailed observations or accounts rather than numerical evidence [43]. As such, the intent of qualitative studies is to understand the phenomenon in depth, rather than to generalize the findings. Although the findings of this study may not be generalizable, they still hold immense value. The intent of this study was never to generalize the findings, but the processes and methods used in this study may be mirrored in future studies to cocreate the principles of compassionate virtual care for other immigrant populations. Common challenges experienced by immigrant populations participating in research studies include scheduling conflicts and lack of transportation [44]. Given these barriers, conducting in-person interviews with some participants may be challenging. Therefore, Zoom will be used as an alternative method for data collection. Trust-related barriers may also pose another potential limitation in this study involving South Asian immigrants [44]. Establishing rapport with participants will be of utmost importance for the interviewer [44].

### Future Directions

#### Practice

With the rapid incentivization to use virtual care services following the onset of the COVID-19 pandemic [18], major aspects of compassionate care provision were lost, and a significant decrease was observed in the quality of health care [45]. In line with this, our findings will ensure that compassionate care for this population can be applied in its entirety. The collaborative approach taken to cocreate these principles is a starting point for developing more compassionate health care practices, as mutual exchange and inclusion are often appreciated by patients [45]. Additionally, our findings can be used to mitigate the disparities in other aspects of compassionate care, such as patient autonomy and security, as well as the provision of holistic health care options [45,46]. It is important that the dearth of knowledge on compassionate virtual care for South Asian communities be addressed and improved. Following this, interventions can be extended to other populations who face disadvantages in receiving compassionate virtual care.

#### Research

To our knowledge, this is the first research proposal to specifically examine barriers to accessing and using compassionate virtual care services among South Asian populations [4,6,10,47]. Therefore, this is a baseline study that will direct future interventions to effectively implement compassionate virtual care among equity-deserving groups. Using the established principles of compassionate care, cocreated with South Asian populations, future research initiatives can direct their efforts toward developing solutions for these disparities and improving virtual health care inequities [45]. Furthermore, this research paper and its collaborative methods can be replicated for Canada’s other immigrant and/or racialized populations; it will highlight the often-homogenized experiences of immigrant populations within Canada with nuance [48-51] rather than generalizing them.

#### Educating Health Care Providers

There is a lack of knowledge of compassionate care practices within the Canadian health care education system [52]. This paper and its understanding of the principles of compassionate care can be used when discerning what areas are in need of better education and training for health care professionals. Sinclair et al [52] identified the complexity of compassion as a potential inhibitor to the provision of compassionate care. The varying and individualized nature of the topic often results in only certain aspects of compassion being addressed, rather than the concept as a whole, leading to inconsistencies in the quality of care provided to patients [52]. The same study found that health care providers whose educational curricula emphasized compassionate care demonstrated a greater ability to relate to and understand their patients, ultimately improving patient outcomes [52]. Evidently, this paper emphasizes the need to adopt such curricula and prioritize the instruction of compassionate care to health professionals [52,53].

### Knowledge Dissemination and Mobilization

We have developed a multifaceted knowledge dissemination and mobilization plan. Although findings will be disseminated through traditional channels, including academic publications and presentations, we will also engage in knowledge mobilization activities with our community and health system partners. This will include conducting knowledge mobilization events with them, preparing infographics in lay language, and delivering presentations and webinars for community members and health system organizations.

### Conclusions

There is a significant gap in the literature regarding South Asian immigrants’ access to and use of virtual care, as well as their perceptions of compassionate virtual care. This study will address an important gap in the existing body of research and serve as a foundation for promoting compassionate virtual care. To achieve this, compassionate, equitable, trusting, and productive patient–health care professional relationships within the context of virtual care will be explored through one-on-one qualitative interviews with South Asian patients. Additionally, principles of compassionate and equitable virtual care will be cocreated through collective virtual meetings with participants. It is anticipated that the collaborative approach taken to cocreate the principles of compassionate and equitable virtual care will serve as a starting point for developing more compassionate health care practices. Moreover, the results can direct future research to effectively implement compassionate virtual care among equity-deserving groups. Finally, understanding of the principles of compassionate care can be used when discerning what areas are in need of better education and training for health care professionals. On the basis of the anticipated outcomes, future research will be conducted to apply the identified principles in practice and test their effectiveness with South Asian immigrants in a culturally safe manner.

## Appendix

Multimedia Appendix 1 Peer review report from the AMS Fellowship in Compassion and Artificial Intelligence.

## Abbreviations

COREQ: Consolidated Criteria for Reporting Qualitative Research
HEIA-DH: Health Equity Impact Assessment-Digital Health supplement
ICA: inductive content analysis

## Data Availability

Data sharing is not applicable to this paper as no datasets were generated or analyzed during this study.

## References

[1] Jedwab J (2011). Multiculturalism. The Canadian Encyclopedia.

[2] Census of population. Government of Canada.

[3] Asian heritage month. Government of Canada.

[4] Population projections on immigration and diversity: interactive dashboard. Government of Canada.

[5] Tsai PL, Ghahari S (2023). Immigrants' experience of health care access in Canada: a recent scoping review. J Immigr Minor Health.

[6] Turin TC, Rashid R, Ferdous M, Naeem I, Rumana N, Rahman A, Rahman N, Lasker M (2020). Perceived barriers and primary care access experiences among immigrant Bangladeshi men in Canada. Fam Med Community Health.

[7] Chowdhury D, Tong C, Lopez K, Neiterman E, Stolee P (2024). "When in Rome…": structural determinants impacting healthcare access, health outcomes, and well-being of South Asian older adults in Ontario using a multilingual qualitative approach. Front Public Health.

[8] Stubbe DE (2020). Practicing cultural competence and cultural humility in the care of diverse patients. Focus (Am Psychiatr Publ).

[9] Reddy P, Chaudhary K, Hussein S (2023). A digital literacy model to narrow the digital literacy skills gap. Heliyon.

[10] Aldosari N, Ahmed S, McDermott J, Stanmore E (2023). The use of digital health by South Asian communities: scoping review. J Med Internet Res.

[11] Hyman A, Stacy E, Mohsin H, Atkinson K, Stewart K, Novak Lauscher H, Ho K (2022). Barriers and facilitators to accessing digital health tools faced by South Asian Canadians in Surrey, British Columbia: community-based participatory action exploration using photovoice. J Med Internet Res.

[12] Perceived usability. ScienceDirect.

[13] The expansion of virtual care in Canada: New Data and Information Internet.

[14] Wosik J, Fudim M, Cameron B, Gellad ZF, Cho A, Phinney D, Curtis S, Roman M, Poon EG, Ferranti J, Katz JN, Tcheng J (2020). Telehealth transformation: COVID-19 and the rise of virtual care. J Am Med Inform Assoc.

[15] Shaver J (2022). The state of telehealth before and after the COVID-19 pandemic. Prim Care.

[16] Webster P (2020). Virtual health care in the era of COVID-19. Lancet.

[17] Bhatia RS, Chu C, Pang A, Tadrous M, Stamenova V, Cram P (2021). Virtual care use before and during the COVID-19 pandemic: a repeated cross-sectional study. CMAJ Open.

[18] Virtual care in Canada. Canadian Institute for Health Information.

[19] About TELUS health. TELUS.

[20] Brual J, Chu C, Fang J, Fleury C, Stamenova V, Bhattacharyya O, Tadrous M (2023). Virtual care use among older immigrant adults in Ontario, Canada during the COVID-19 pandemic: a repeated cross-sectional analysis. PLOS Digit Health.

[21] Sierra-Heredia C, Tayyar E, Bozorgi Y, Thakore P, Hagos S, Carrillo R, Machado S, Peterson S, Goldenberg S, Wiedmeyer ML, Lavergne MR (2024). Growing inequities by immigration group among older adults: population-based analysis of access to primary care and return to in-person visits during the COVID-19 pandemic in British Columbia, Canada. BMC Prim Care.

[22] Nigusie A, Endehabtu BF, Angaw DA, Teklu A, Mekonnen ZA, Feletto M, Assan A, Samuel A, Sheikh K, Tilahun B (2022). Status of compassionate, respectful, and caring health service delivery: scoping review. JMIR Hum Factors.

[23] Jemal K, Hailu D, Mekonnen M, Tesfa B, Bekele K, Kinati T (2023). The importance of compassion and respectful care for the health workforce: a mixed-methods study. Z Gesundh Wiss.

[24] Singh P, King-Shier K, Sinclair S (2020). South Asian patients' perceptions and experiences of compassion in healthcare. Ethn Health.

[25] Guetterman TC, Koptyra E, Ritchie O, Marquis LB, Kadri R, Laurie A, Vydiswaran VV, Li J, Brown LK, Veinot TC, Buis LR (2025). Equity in virtual care: a mixed methods study of perspectives from physicians. J Telemed Telecare.

[26] Osmanlliu E (2025). Virtual emergency departments: enabling accessible and compassionate care through inclusive technology. CJEM.

[27] Hodges BD, Paech G, Bennett J (2020). Without Compassion, There Is No Healthcare: Leading with Care in a Technological Age.

[28] Vaughn LM, Jacquez F (2020). Participatory research methods – choice points in the research process. J Particip Res Methods.

[29] Flanagan JM, Beck CT (2024). Polit & Beck's Nursing Research.

[30] Johnson C, Wilhelmsson S, Börjeson S, Lindberg M (2015). Improvement of communication and interpersonal competence in telenursing--development of a self-assessment tool. J Clin Nurs.

[31] Crawford A (2022). Health Equity Impact Assessment (HEIA): digital health supplement. Centre for Addiction and Mental Health.

[32] Keller M, Plishka C, King M, Gunderson J, Stobart CA, Dunn K, Haver CR (2020). The creation of a tool to measure engagement in patient-oriented research. Healthc Q.

[33] Strategy for patient-oriented research - patient engagement framework. Canadian Institutes of Health Research.

[34] Pearce G, Magee P (2024). Co-creation solutions and the three Co’s framework for applying co-creation. Health Educ.

[35] Risling TL, Risling DE (2020). Advancing nursing participation in user-centred design. J Res Nurs.

[36] Elliott RK Jr, Timulak L (2021). Essentials of Descriptive-Interpretive Qualitative Research: A Generic Approach.

[37] Thorne S (2020). Untangling the misleading message around saturation in qualitative nursing studies. Nurse Author Ed.

[38] Malterud K, Siersma VD, Guassora AD (2016). Sample size in qualitative interview studies: guided by information power. Qual Health Res.

[39] Mihas P (2019). Qualitative data analysis. Oxford Research Encyclopedia of Education.

[40] Vears DF, Gillam L (2022). Inductive content analysis: a guide for beginning qualitative researchers. Focus Health Prof Educ.

[41] Malenfant S, Jaggi P, Hayden KA, Sinclair S (2022). Compassion in healthcare: an updated scoping review of the literature. BMC Palliat Care.

[42] Greenhalgh T, Jackson C, Shaw S, Janamian T (2016). Achieving research impact through co-creation in community-based health services: literature review and case study. Milbank Q.

[43] Tenny S, Brannan JM, Brannan GD (2026). Qualitative study. StatPearls [Internet].

[44] De La Rosa M, Babino R, Rosario A, Martinez NV, Aijaz L (2012). Challenges and strategies in recruiting, interviewing, and retaining recent Latino immigrants in substance abuse and HIV epidemiologic studies. Am J Addict.

[45] Wu K, Dang Nguyen M, Rouleau G, Azavedo R, Srinivasan D, Desveaux L (2025). Understanding how virtual care has shifted primary care interactions and patient experience: a qualitative analysis. J Telemed Telecare.

[46] MacPherson M (2023). Immigrant, refugee, and Indigenous Canadians' experiences with virtual health care services: rapid review. JMIR Hum Factors.

[47] Levesque JF, Harris MF, Russell G (2013). Patient-centred access to health care: conceptualising access at the interface of health systems and populations. Int J Equity Health.

[48] Lin SL (2021). Access to health care among racialised immigrants to Canada in later life: a theoretical and empirical synthesis. Ageing Soc.

[49] Comeau D, Johnson C, Bouhamdani N (2023). Review of current 2SLGBTQIA+ inequities in the Canadian health care system. Front Public Health.

[50] Borras AM (2023). The challenge of exposing and ending health inequalities through social and policy change: Canadian experiences. Int J Soc Determinants Health Health Serv.

[51] Kehoe MacLeod K, Flores KN, Chandra K (2023). Identifying facilitators and barriers to integrated and equitable care for community-dwelling older adults with high emergency department use from historically marginalized groups. Int J Equity Health.

[52] Sinclair S, Kondejewski J, Jaggi P, Dennett L, Roze des Ordons AL, Hack TF (2021). What is the state of compassion education? A systematic review of compassion training in health care. Acad Med.

[53] Kemp J, Zhang T, Inglis F, Wiljer D, Sockalingam S, Crawford A, Lo B, Charow R, Munnery M, Singh Takhar S, Strudwick G (2020). Delivery of compassionate mental health care in a digital technology-driven age: scoping review. J Med Internet Res.

