# Supplementary material for: Cocreating the Principles of Compassionate Virtual Care With South Asian Immigrants: Protocol for a Qualitative Study
Source: JMIR Res Protoc. 2026 Aug 27;15:e103397. doi: 10.2196/103397 (PMC13563030; doi:10.2196/103397)
Supplement: Supplementary file 1 [file resprot_v15i1e103397_app1.pdf]

advancing innovative healthcare with compassion at its core

Dr. Saleema Allana
Assistant Professor
University of Western Ontario
By Email: sallana5@uwo.ca

Thursday, June 15, 2023

Re: Award Notification Letter
AMS Fellowship in Compassion and Artificial Intelligence

Dear Dr. Allana,

Thank you for your interest in the AMS Fellowship in Compassion and Artificial Intelligence. I am pleased to inform you that your application entitled ***Co-creating Principles of Compassionate Virtual Care (VC) for South Asian Immigrants: A Patient-and-Community-Partnered Research Project*** has been selected for funding. The principal focus of the AMS Fellowship in Compassion and AI award is to advance the goals of AMS Healthcare as outlined in your application submitted for funding. You will find reviewer comments in Appendix A of this letter. Details of the award are as follows:

**Funding**	$75,000
**Funding Start**	September 2023
**% of Time Allocated to Fellowship**	50%
**Name of Supervisor/Mentor**	Victoria Smye/Richard Booth/Alison Crawford/Tracie Risling/Javeed Sukhera

To activate this award, the following actions must be completed, and the required forms submitted to the AMS Healthcare by **5:00 pm** EDT on Thursday, August 3rd, 2023:

1. **Review and sign this Award Notification Letter. Your signature indicates your acceptance of the AMS Fellowship in Compassion and Artificial Intelligence Award, and that you have reviewed the attached AMS Healthcare Grant/Award Holder's Guide (2023)**
2. **Complete the attached EFT Enrollment Form**
3. **Attach a copy of your Institutional Banking Information Letter OR void cheque.**
4. **You will also receive a separate email and be asked to complete and return a communications package including brief biography, photo, etc.**

The AMS Healthcare Grant/Award Holder's Guide (2023) indicates the requirement for you to attend an in-person residency in Toronto. Please note that this is scheduled for October 18-20, 2023. The fellowship programming will occur over a 12-month period (September 2023 - August 2024). While it is hoped that fellows will complete their projects and learning during that period, accommodations can be made should additional time be required. Please contact Jocelyn Bennett, AMS Director Programs (jocelyn.bennett@amshealthcare.ca) if you have any questions regarding any of these requirements.

AMS | advancing innovative healthcare with compassion at its core

Failure to complete any of these actions by the prescribed deadline will indicate your rejection of the award. Funds will subsequently be allocated to alternative project(s).

We will be formally announcing the AMS Fellowship recipients in September 2023. We will be coordinating communication with your organization, and we ask that you do not announce, or publicly share this information until this time. You may inform your mentors, supervisors, collaborators, friends, and family prior to that date.

Congratulations on being selected as an AMS Fellow in Compassion and Artificial Intelligence.

Regards,

Helen Angus
CEO, AMS Healthcare

I accept the AMS Fellowship in Compassion and AI Award and agree to the terms and conditions as outlined in the AMS Healthcare Grant/Award Holder's Guide.

	Dr. Saleema Allana	June 22, 2023
Signature of Award Recipient	Print Name	Date
Authorized Signature of Supporting Institution	Print Name	Date

Please scan and email signed copies of this signed *Acceptance Letter* and completed *EFT Enrollment Form* to Jocelyn.bennett@amshealthcare.ca no later than 5:00 pm Thursday, August 3rd, 2023.

Please direct any questions to: Jocelyn Bennett, AMS Director Programs
Jocelyn.bennett@amshealthcare.ca

APPENDIX A – Reviewer Comments

"Dr. Saleema Allana is an Assistant Professor at the University of Western Ontario.

The project seeks to understand the specific needs and principles of compassionate virtual care (VC) for South-Asian (SA) immigrants. The proposal has well defined connections to AMS mission vision and values with strong relevance for current context.

This applicant has the capacity and experience to be the right professional to be successfully with this project (digital health technology). She does express her goal of compassionate VC for SA immigrants as ultimately, she wants to be a leader in compassion and digital health technology. Her expertise and resume certainly confirm her capacity and being relevant for this fellowship. She has the required background to undertake the project and has been recognized by her own institution and Tri-council agencies with immigrants.

The host institution, Western University, will support this fellowship with contributions described in the letters of support. The faculty has provided her with the environment to be able to carry this project and she is well supported by her supervisors and mentors, each of whom play a significant and specific role in her activities. She has support in recruiting participants for her qualitative approach.

The applicant noted issues with immigrants and inequities in access and use VC healthcare. She wants to explore what is compassion, equity, trust and productive patient-provider relationships means for SA immigrants in the context of VC. She wants to collaborate with SA to co-create the principles of compassionate VC specific to their needs with the ultimate goal to advance equitable access to VC and increase patient satisfaction and care outcomes.

The activities are well defined and appropriate methods used to address an important current gap in knowledge. The reviewers noted that the proposal only mentions ""satisfaction"" in the lay summary. So, her focus should be related to the inequities that seem to be the main barrier. Her planned activities are certainly realistic as she will involve the target groups (SA and clinicians) in the last 2 phases. She also identified issues with dissemination, but her activities will certainly create some ""food for thought"". It remains unclear what sector of virtual care delivery will be of interest (specialist, primary, etc...). The reviewers also questioned whether the proposed design (critical ethnographic approaches) clearly aligns to the methods outlined (e.g. observations are not included). The activities in phases 1 and 3 are not as well described (so unclear what will take place). Finally, there is a bit of a concern that there are others working in this space (including some recent scoping reviews) that have not been cited - the proposal needs better grounding in the emerging literature."
